# Stimulated Raman adiabatic passage in a three-level superconducting circuit

**DOI:** 10.1038/ncomms10628

**Published:** 2016-02-23

**Authors:** K. S. Kumar, A. Vepsäläinen, S. Danilin, G. S. Paraoanu

**Affiliations:** 1 Low Temperature Laboratory, Department of Applied Physics, Aalto University School of Science, PO Box 15100, Aalto FI-00076, Finland

## Abstract

The adiabatic manipulation of quantum states is a powerful technique that opened up new directions in quantum engineering—enabling tests of fundamental concepts such as geometrical phases and topological transitions, and holding the promise of alternative models of quantum computation. Here we benchmark the stimulated Raman adiabatic passage for circuit quantum electrodynamics by employing the first three levels of a transmon qubit. In this ladder configuration, we demonstrate a population transfer efficiency >80% between the ground state and the second excited state using two adiabatic Gaussian-shaped control microwave pulses. By doing quantum tomography at successive moments during the Raman pulses, we investigate the transfer of the population in time domain. Furthermore, we show that this protocol can be reversed by applying a third adiabatic pulse, we study a hybrid nondiabatic–adiabatic sequence, and we present experimental results for a quasi-degenerate intermediate level.

## Abstract (web summary)

The precise control and manipulation of the states of a multi-level quantum system are fundamental for quantum information processing. Here, the authors demonstrate the robust adiabatic manipulation of the quantum states of a superconducting circuit via stimulated Raman adiabatic passage.

## Graphical abstract

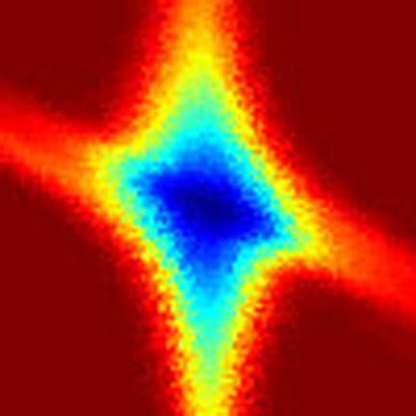

The precise control and manipulation of the states of multilevel quantum systems is a key requirement in quantum information processing. Using multilevel systems as the basic components of quantum processors instead of the standard two-level qubit brings along the benefit of an extended Hilbert space, thus ultimately reducing the number of circuit elements required to perform a computational task^1^,^2^. Superconducting qubits can be operated as multilevel systems, and to date fundamental effects related to three-level artificial atoms irradiated with continuous microwave fields—for example, the Autler–Townes effect^3^,^4^,^5^, coherent population trapping^6^ and electromagnetically induced transparency^7^—have been reported. An additional powerful concept is that of adiabatic quantum control, a key component in several proposed models of quantum computation^8^,^9^,^10^,^11^,^12^,^13^, which in recent times led to the measurement of the Berry phase^14^ and its nonabelian generalization^15^, and to the observation of topological transitions^16^.

The main idea in this paper is to employ a superconducting circuit with energy levels in the ladder configuration and irradiated with two microwave fields to demonstrate a fundamental quantum-mechanical process called stimulated Raman adiabatic passage (STIRAP)^17^,^18^: the transfer of population between two states |0〉 and |2〉 without populating the intermediate state |1〉. The absence of population in the intermediate state is ensured by a two-photon destructive interference on the intermediate level that creates a dark state, while the adiabatic manipulation of the amplitudes of the control fields rotates the dark state from the initial state |0〉 to the target state |2〉. The STIRAP technique was first demonstrated with sodium dimer molecular beams^19^ and applied since then in many contexts in atomic physics. It has remained a topic of active research^20^, and in recent years unexpected connections and analogies have been found. Recently, there have been proposals on designing superconducting circuits with similar energy structure as the atoms, where the adiabatic transfer of population could be observed^21^,^22^,^23^,^24^. However, due to the present pre-eminence of the transmon as the building block of future superconducting quantum processors, it would be most relevant to demonstrate this technique on a typical device of this kind. The transmon^25^ is a capacitively shunted split Cooper pair box coupled dispersively with an electromagnetic coplanar waveguide cavity^26^, which allows the non-demolition measurement of its quantum state by monitoring the near-resonant response of the cavity^27^. With this device it is not possible to transfer directly the population between the ground state |0〉 and the second excited state |2〉 due to the fact that the electric dipole momentum between these two states is vanishingly small. Thus, this transfer must be done by first populating the first excited state |1〉 using a *π*-pulse applied to the 0–1 transition, then transferring this population to the second excited state |2〉 using another *π*-pulse applied to the 1–2 transition. This is called an intuitive sequence, and requires a precise control of the pulse sequence, as well as of the frequencies of the microwaves. In contrast, with STIRAP the population transfer is based on adiabatically varying control microwave fields, applied in a counter-intuitive order, that is, with the first pulse driving the 1–2 transition and the second pulse, having a suitable overlap with the first one, driving the 0–1 transition. There is no population in the intermediate state during the process, therefore the decay associated with the intermediate state does not matter.

In the following, we aim at benchmarking the STIRAP protocol under realistic conditions—with a transmon that has finite decoherence times associated to the excited states. Our results show that the adiabatic nature of the population transfer makes the protocol very robust to the timing and the amplitudes of the pulses. Ideally, to ensure adiabaticity one should use long pulses. However, we find that this is not a strong restriction, and the protocol can be run relatively fast using shorter pulses. We also show that this protocol can be reversed by applying a third adiabatic pulse. Furthermore, we study the effect of applying STIRAP to a superposition between the ground and the first excited state, and we study experimentally the adiabatic Raman sequence in the case of a split intermediate level.

## Results

### Theoretical model

We consider a transmon (Supplementary Fig. 1) driven by two microwave pulses of amplitudes Ω_01_(*t*) and Ω_12_(*t*) with frequencies 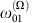 and 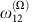, respectively, possibly slightly detuned from the corresponding qubit transition frequencies *ω*_01_ and *ω*_12_. In the dispersive regime and in the rotating wave approximation with respect to the two driving tones (see Supplementary Note 1), the effective three-level Hamiltonian of the driven transmon reads^3^,^5^,^15^


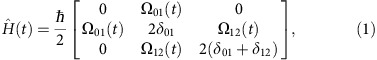


where the detunings 
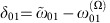
 and 
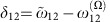
 are defined with respect to the Lamb shift renormalized transition frequencies of the transmon 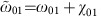 and 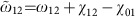, and for simplicity Ω_01_ and Ω_12_ are taken real. The rates *χ*_01_ and *χ*_12_ are the a.c. Stark shifts of the corresponding transitions. If the two-photon resonant condition *δ*_01_+*δ*_12_=0 is satisfied, the Hamiltonian equation (1) has a zero-eigenvalue eigenstate called dark state, |D〉=cos Θ|0〉−sin Θ|2〉 (see Methods), with Θ defined by tan Θ=Ω_01_(*t*)/Ω_12_(*t*). Due to the adiabatic theorem, by slowly tuning the drive amplitudes Ω_01_(*t*) and Ω_12_(*t*), such that tan Θ goes from zero to infinity, the population transfer from the ground state to the second excited state can be realized.

In the experiment we work with Gaussian pulses, parametrized as


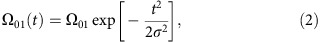



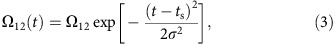


where *t*_s_ is the time separation between the maxima of the pulses, *σ* is the width of the pulse, and Ω_01_ and Ω_12_ are the amplitudes. We choose equal-amplitude pulses, Ω_01_≈Ω_12_≈Ω. The general condition for the adiabatic following^17^,^18^ is given by 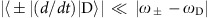. Using equation (2) and performing a time integration (Supplementary Note 2), we obtain the global condition of adiabaticity for Gaussian pulses,


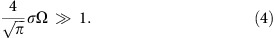


This global adiabatic condition shows that the transfer efficiency can be improved by making the pulses longer, which is in turn limited by the decoherence of the transmon. In principle, if a high transfer efficiency is desired, the shape of the pulses could be further optimized^28^,^29^.

### Benchmarking the STIRAP sequence for the transmon

From the spectroscopy data, as well as from the change of the Rabi frequency as a function of the detuning, we determine the Lamb shift renormalized transition frequencies of the transmon 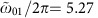 GHz and 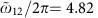 GHz. The decay and pure dephasing rates extracted from standard characterization measurements were Γ_10_=2.4 MHz, Γ_21_=5.3 MHz, 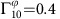 MHz, 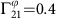 MHz and 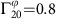 MHz, while the Gaussian pulses were calibrated to Ω_01_/2*π*=43.4 MHz and Ω_12_/2*π*=38.2 MHz, with widths *σ*=43 ns for both pulses (see Methods). This choice satisfies the adiabatic condition in equation (4) by a factor of 4.

**Figure 1:**
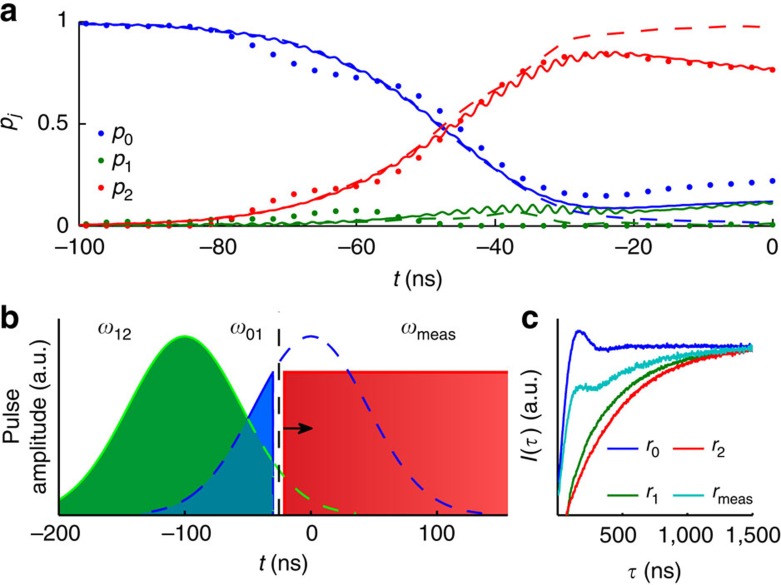
Quantum tomography of the STIRAP process in a transmon. (**a**) The state of the quantum system at different times during the adiabatic population transfer at fixed pulse separation *t*_*s*_=−92 ns. The dots represent experimental results, the dashed line is a simulation of the three-level unitary evolution, and the continous lines show the simulation with decoherence included (see Methods). During the time evolution the intermediate state |1〉 remains almost unpopulated. (**b**) Schematic of the pulse sequence during the STIRAP. (**c**) The population of the system at every time *t* is calculated from the cavity response 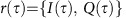 (only 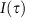 shown here), and the measurement data (cyan) is compared with the calibration data for the known states |0〉 (blue), |1〉 (green) and |2〉 (red).

Figure 1a demonstrates the STIRAP protocol for our system, with the population of the state |2〉 reaching a maximum value during the pulse overlap and then slowly decreasing at large timescales due to the intrinsic decay of the second excited state. The pulse sequence is presented in Fig. 1b. The results of the quantum tomography calibration for the three levels are shown schematically in Fig. 1c. The traces are obtained by a homodyne measurement of the cavity response 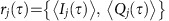, where *j*=0, 1 and 2 for the reference states |0〉, |1〉 and |2〉, respectively, which are prepared using fast microwave pulses applied to the 0–1 and 1–2 transitions (Supplementary Note 3). Here *I*_*j*_ and *Q*_*j*_ are the in-phase and the quadrature fields, respectively. For an arbitrary state *ρ*(*t*) at time *t* during the STIRAP protocol, the cavity response is a linear combination^30^ 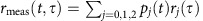, where *p*_*j*_(*t*)=T*r*[*ρ*(*t*)|*j*〉〈*j*|] is the population of state |*j*〉. We extract the populations *p*_*j*_(*t*) using the Levenberg–Marquardt algorithm. We also note that the tomography method presented here includes interrupting the STIRAP protocol before the full population is transferred, a protocol called fractional STIRAP^31^.

From the data, we can see that the population in the second excited state reaches about 83% and the intermediate state gets slowly populated due to the decay |2〉→|1〉. The main sources of nonideality are the decoherence of the upper levels, overshooting and undershooting in the shape of the pulses that effectively reach the sample, as well as imperfections in the timing of the short pulses used for calibration. We note as well that the STIRAP transfer occurs in fact quite fast, predominantly in the overlap region: the optimal value is obtained not too far from the beginning of the *ω*_01_ pulse. This means that the duration of the STIRAP process can be shortened by interrupting it earlier (with correspondingly shaped pulses) without too big penalties on the fidelity of state transfer, thereby reducing the overall gate length.

**Figure 2:**
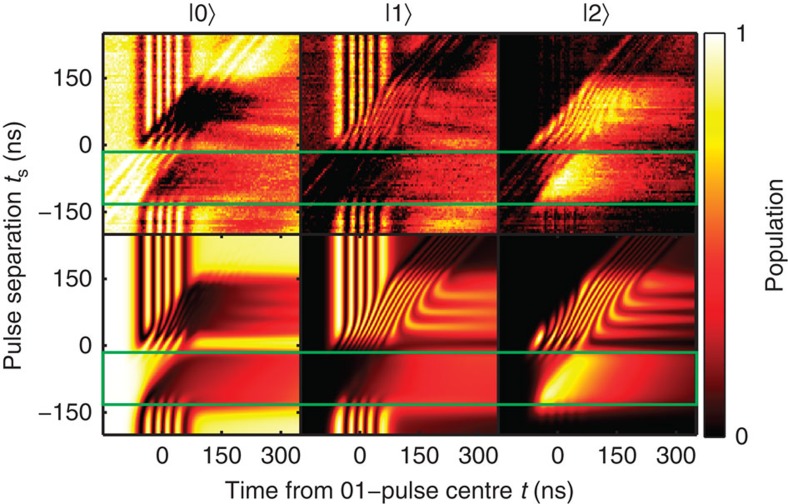
STIRAP sequence at different pulse separations. The upper figures show the experimental results for the time evolution of states |0〉,|1〉 and |2〉, whereas the lower row shows the corresponding simulation results, calculated using Lindblad's master equation for a multilevel Jaynes–Cummings Hamiltonian (see Methods). The separation time *t*_s_=0 corresponds to a complete overlapping of the pulses. The STIRAP sequence (data inside the green rectangles) occurs at negative values of the pulse separation, while positive values of the pulse separation correspond to the intuitive sequence.

Next, we present a complete experimental investigation of this effect, together with numerical simulations. By varying the time separation between the two pulses, one can study the counterintuitive as well as the intuitive sequence. In Fig. 2, we present the occupation probability of each of the states |0〉, |1〉 and |2〉 at different measurement times (horizontal axis), measured from the peak of the 0–1 pulse. The pulse separation (vertical axis) is the time interval between the peak of the 1–2 pulse and the peak of the 0–1 pulse. Thus, zero pulse separation means complete pulse overlap, negative values of the pulse separation refer to the counter-intuitive pulse sequence (*ω*_12_ is applied before *ω*_01_ pulse), whereas positive values indicate that the pulses are sent in the intuitive order. The lower panels of Fig. 2 show the corresponding simulation result using Lindblad's master equation (see equation (7) in Methods). From the experimental data we find that the optimal value for the pulse separation is ≈−90 ns, in close agreement with the result in ref. 28 and our own simulations. Importantly, one notices that for STIRAP the transfer efficiency is high and a relatively slowly varying function of the pulse overlap on a plateau extending from about −120 to about −80 ns, which demonstrates that accurate optimization of the pulse overlap is not required. In principle, population transfer can also be realized using the intuitive-pulse sequence, but in this case the method is sensitive to the timing of the pulses, as can be seen from the oscillations present at positive pulse separation in Fig. 2. Clearly, the stability plateaus of the STIRAP do not form in the case of the intuitive sequence. Moreover, in the presence of decoherence it is harder to achieve transfer fidelities as high as with counter-intuitive pulse sequence, because the pulse sequence cannot be reliably interrupted until it is completely finished. This robustness against errors in the timing of the pulses demonstrated here is one of the most significant benefits of STIRAP over non-adiabatic methods of population transfer.

**Figure 3:**
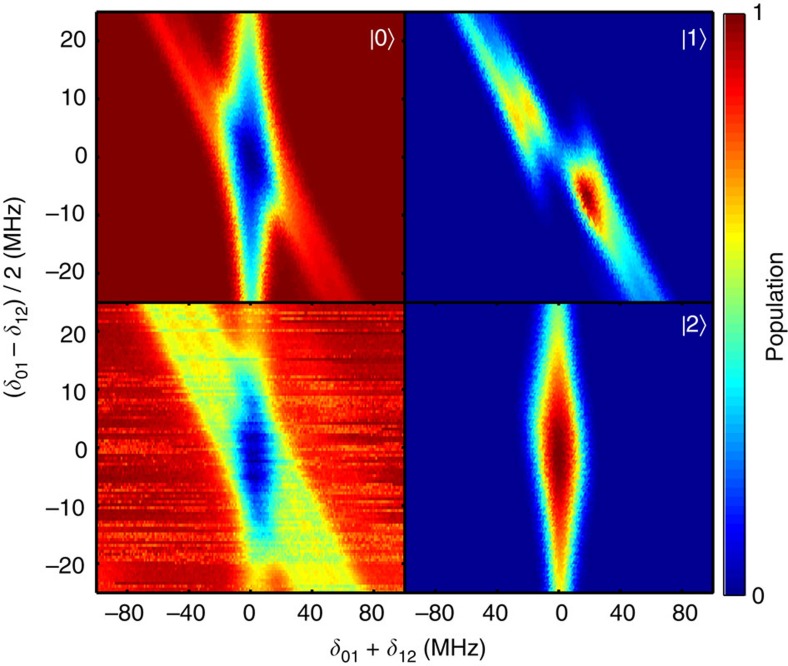
STIRAP dependence on single-photon and two-photon detunings. Here the plot in the lower-left corner is the measured ground state population, while the corresponding simulation results are shown in the other three plots. The offset from zero of the sum of the detunings *δ*_01_+*δ*_12_ (horizontal axis) corresponds to the violation of the two-photon resonance condition, to which the transfer is mostly sensitive. Nonzero values of the half-difference of the detunings (*δ*_01_−*δ*_12_)/2 (vertical axis) correspond to the violation of the single-photon detuning, against which the system is robust. The data were taken for a pulse separation *t*_s_=−90 ns.

STIRAP is also quite robust with respect to frequency shifts of the intermediate state: indeed, from Fig. 3 one can see that if the two-photon resonance condition *δ*_01_+*δ*_12_=0 is not violated, the transfer is possible with relatively large single-photon detunings. Finally, STIRAP is not very sensitive to the widths of the pulses: we have tested *σ*=40, 60 and 70 ns, and obtained similarly large values for the transfer efficiency. We find that when decreasing the pulse width below these values the adiabaticity condition is no longer fulfilled and oscillations start to appear, while if it is too large the effect of decay becomes significant.

### Hybrid nonadiabatic–adiabatic sequence

**Figure 4:**
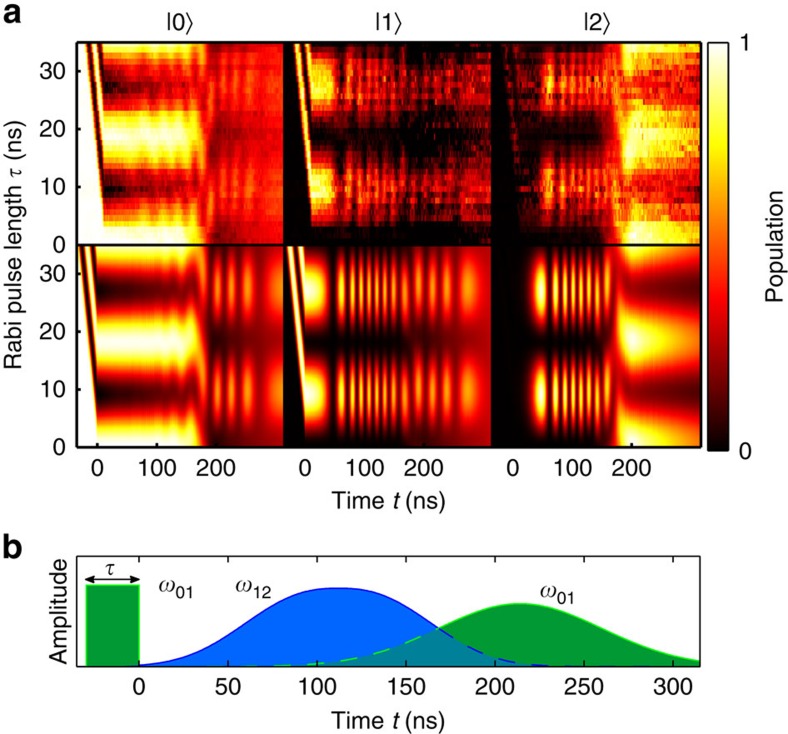
Hybrid sequence. A Rabi pulse before the STIRAP pulse sequence creates a superposition between the ground state and the intermediate state |1〉, which results in a reduction of the population of level |2〉 at the end of the entire sequence. (**a**) The upper row shows the measurement results for the three states, while the corresponding simulation results are shown on the lower row. (**b**) The pulse sequence used to generate the measurement results. The separation between the adiabatic pulses was *t*_s_=−92 ns.

In a quantum processor based on qutrits, it might be more efficient to use combinations of fast pulses and adiabatic pulses to prepare three-level target states, with certain fidelities or to realize hybrid nonadiabatic–adiabatic gates. With superconducting qutrits, this has been achieved so far with a sequence of fast (nonandiabatic) pulses^32^, as well as with a technique based on a single multi-tone excitation pulse^33^. This motivates us to study hybrid pulses, where, for example, a fast pulse is applied to the 0–1 transition before a STIRAP sequence, see Fig. 4. This corresponds to starting the adiabatic population transfer protocol from a superposition between the ground state and the first excited state. During the evolution of the system, this results in a superposition between the dark state and the intermediate state, as shown in Fig. 4. Towards the end of the sequence, the population on level 2 reaches its maximum value, which is determined by the amplitude and duration of the nonadiabatic 0–1 pulse (see Supplementary Note 4). The populations of states 0 and 1 oscillate due to the Rabi coupling provided by the last (adiabatic) *ω*_01_ pulse, which determines their final values (Supplementary Fig. 2). As a result, we can create superpositions of all three states by adjusting only the strength of the *ω*_01_ pulses and without violating the adiabaticity condition.

### Reversal of STIRAP

**Figure 5:**
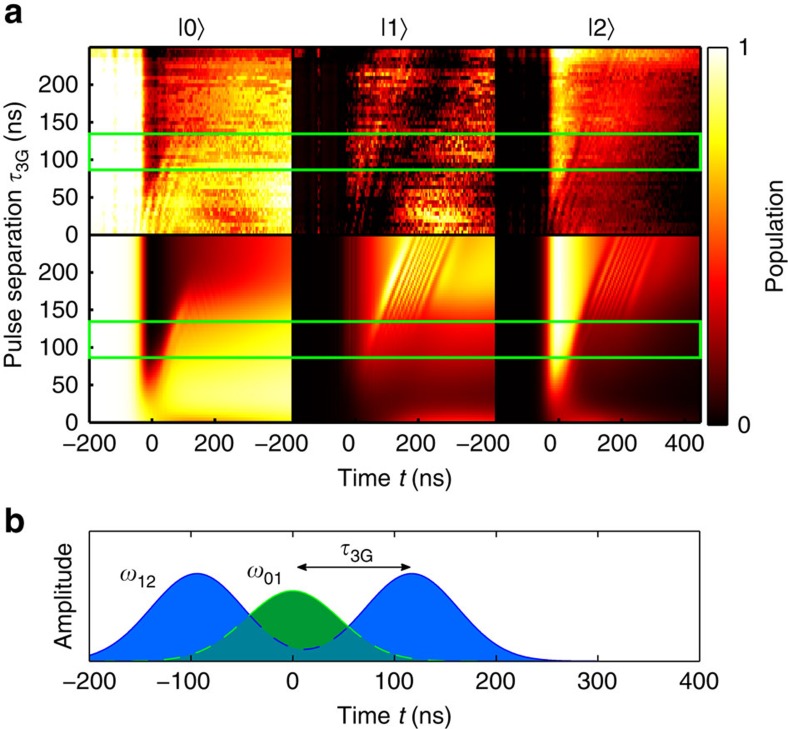
STIRAP reversal. Three adiabatic pulses are used to first excite the system to the second excited state and then bring it back to the ground state. (**a**) Time evolution of the state (with the green rectangles indicating the region where the process is the most efficient): the upper row shows the experimental data, while the lower row is the simulation. (**b**) Schematic of the applied pulse sequence, with a fixed separation of *t*_s_=−92 ns between the first two pulses, and a variable time 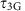 between the second and the third Gaussian pulse.

In Fig. 5a, we show how the STIRAP can be reversed by applying one additional Gaussian pulse at the 1–2 transition, demonstrating that STIRAP can be used to transfer population from state |2〉 back to the ground state. The idea is to first transfer the population to the second excited state using STIRAP, and then apply an inverted STIRAP sequence (the 0–1 pulse is followed by a 1–2 pulse) on the system in state |2〉 to bring it back to the ground state. The reversal of the STIRAP is essential in creating adiabatic gates such as the single-qubit phase gate^34^ (see Supplementary Note 5).

### STIRAP protocol with a split intermediate state

**Figure 6:**
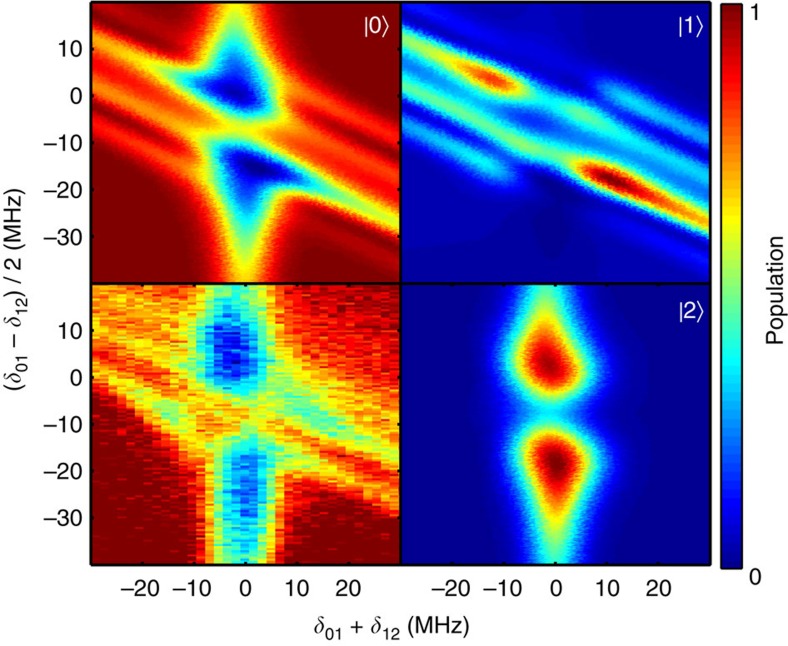
STIRAP with a quasi-degenerate intermediate state. The splitting of the first excited state into two states |1〉 and |1′〉 separated by ∼11.5 MHz suppresses the transfer efficiency when the one-photon detuning is between the split levels. The experimental result for the ground state is shown in the lower-left corner and the corresponding simulations are labelled with |0〉, |1〉 and |2〉. The time separation between the pulses was *t*_s_=−69 ns.

Finally, an interesting situation is that of a quasi-degenerate intermediate state (Supplementary Fig. 3). To realize this degeneracy, we did several thermal cycles of the sample, until we created a defect in the insulating layer of the junctions, observed by the splitting of the spectral line into states |1〉 and |1′〉 (see Supplementary Note 6, Supplementary Figs 4 and 5). The formation of this type of defect has been observed recently in flux qubits as well^35^. In this case an interesting two-blob structure can be seen in the detuning plot, as shown in Fig. 6, where each blob corresponds to the single-photon resonance condition of either |1〉 or |1′〉.

## Discussion

We have demonstrated the adiabatic transfer of population from the ground state |0〉 to the second excited state |2〉 in a three-level transmon circuit. This benchmarks the STIRAP protocol as a valid procedure for quantum information processing applications in circuit quantum electrodynamics (QED). We note that while in the previous continuous-wave experiments on various types of qubits^3^,^4^,^6^,^7^, it was not possible to asses unambiguosly the effect of quantum interference from the spectrocopic data^36^, our experiment establishes it conclusively. We also studied the effect of having a finite initial state population in the dark state, we demonstrated the adiabatic reversal of the STIRAP protocol and we realized the transfer of population also via a quasi-degenerate intermediate state. The main limiting factor is decoherence, which is presently improving fast with new designs and fabrication techniques for superconducting circuits. We find that the process is very resilient to errors in the timing of the pulses and single-photon detunings from the microwave drives.

## Methods

### Three-level effective Hamiltonian

The effective Hamiltonian of the system in a doubly rotating frame and under the rotating-wave approximation takes the matrix form presented in equation (1). If the two-photon detuning *δ*_01_+*δ*_12_ is zero, the eigenvalues of the above Hamiltonian are 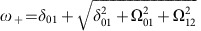, 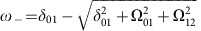 and *ω*_D_=0, with the corresponding eigenvectors


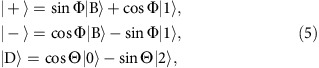


where the zero eigenvalue is called the dark state, and the state orthogonal to it |B〉=sin Θ|0〉+cos Θ|2〉 is called the bright state. The angle Θ is defined by tan Θ=Ω_01_(*t*)/Ω_12_(*t*) and it parametrizes the rotation in the {|0〉, |2〉} subspace (spanned also by the bright and the dark states), while


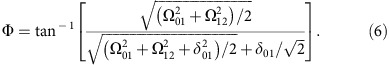


For zero detuning *δ*_01_=0, we have Φ=*π*/4. The adiabatic tuning of the pulse amplitudes ensures that the state of the system follows the |D〉 eigenstate instead of being nonadiabatically excited to either one of the |±〉 eigenstates, which contain contributions from the state |1〉.

### Sample characterization

The transition frequencies of the transmon 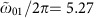 GHz and 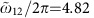 GHz are obtained from the spectroscopy data, as well as from the Chevron patterns of the detuned Rabi oscillations. The measurements of Rabi oscillations on the 1–2 transition are done by first applying a *π*-pulse to the 0–1 transition followed by the Rabi pulse on the 1–2 transition. To obtain the decay rate Γ_10_, we employ a *π*-pulse on the 0–1 transition and measure the population of the level 1 as it decays. To find Γ_12_, we use a *π*-pulse applied to the 0–1 transition, then a *π*-pulse on the 1–2 transition, and we fit the data with a three-level model that includes the decay 2→1 and 1→0. We obtain the energy relaxation rates Γ_10_=2.4 MHz and Γ_21_=5.3 MHz. From Ramsey interference experiments, we get 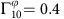 MHz and 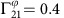 MHz, and following the analysis of pure dephasing in three-level systems^5^, we conclude that the pure dephasing 0–2 is at most 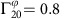 MHz. The Gaussian pulses are created by a high sample rate arbitrary waveform generator followed by mixing with a microwave pulse (see Supplementary Note 1 and Supplementary Fig. 1). They are calibrated using the Rabi data, resulting in Ω_01_/2*π*=43.4 MHz and Ω_12_/2*π*=38.2 MHz, with standard deviation *σ*=43 ns for both pulses. The quantum tomography procedure follows ref. 30 (see Fig. 1c) and the Supplementary Note 3 for details).

### Numerical simulations

For the simulations we use the three-level Lindblad master equation


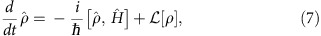


where by employing the notation *σ*_*ij*_=|*i*〉〈*j*| we can write the Lindblad superoperator in the form^5^,^7^


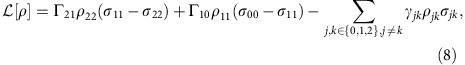


with the off-diagonal decay rates *γ*_*jk*_=*γ*_*kj*_ defined as 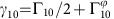, 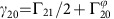 and 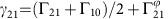. In the simulations, besides the smooth standard STIRAP features, some additional oscillations are present: these are due to the cross-coupling between the driving fields into the other transitions, and as such these oscillations are a consequence of the reduced anharmonicity of the transmon. The matching with the data can be in principle improved by including deformations of the Gaussian pulses as it is seen by the qubit after the propagation through the input lines and sample holder.

## Additional information

**How to cite this article:** Kumar, K. S. *et al*. Stimulated Raman adiabatic passage in a three-level superconducting circuit. *Nat. Commun.* 7:10628 doi: 10.1038/ncomms10628 (2016).

## Supplementary Material

Supplementary Information Supplementary Figures 1-5, Supplementary Notes 1-6 and Supplementary References

## References

[b1] Lanyon B. P. . Simplifying quantum logic using higher-dimensional hilbert spaces. Nat. Phys. 5, 134–140 (2009).

[b2] Ralph T. C., Resch K. & Gilchrist A. Efficient Toffoli gates using qudits. Phys. Rev. A 75, 022313 (2007).

[b3] Sillanpää M. A. . Autler-Townes effect in a superconducting three-level system. Phys. Rev. Lett. 103, 193601 (2009). 20365921 10.1103/PhysRevLett.103.193601

[b4] Baur M. . Measurement of Autler-Townes and Mollow transitions in a strongly driven superconducting qubit. Phys. Rev. Lett. 102, 243602 (2009). 19659005 10.1103/PhysRevLett.102.243602

[b5] Li J. . Decoherence, Autler-Townes effect, and dark states in two-tone driving of a three-level superconducting system. Phys. Rev. B 84, 104527 (2011).

[b6] Kelly W. R. . Direct observation of coherent population trapping in a superconducting artificial atom. Phys. Rev. Lett. 104, 163601 (2010). 20482047 10.1103/PhysRevLett.104.163601

[b7] Abdumalikov A. A. . Electromagnetically induced transparency on a single artificial atom. Phys. Rev. Lett. 104, 193601 (2010). 20866963 10.1103/PhysRevLett.104.193601

[b8] Das A. & Chakrabarti B. K. Quantum annealing and analog quantum computation. Rev. Mod. Phys. 80, 1061–1081 (2008).

[b9] Johnson M. W. . Quantum annealing with manufactured spins. Nature 473, 194–198 (2011). 21562559 10.1038/nature10012

[b10] Boixo S. . Evidence for quantum annealing with more than one hundred qubits. Nat. Phys. 10, 218–224 (2014).

[b11] Sjöqvist E. . Non-adiabatic holonomic quantum computation. New J. Phys. 14, 103035 (2012).

[b12] Zanardi P. & Rasetti M. Holonomic quantum computation. Phys. Lett. A 264, 94–99 (1999).

[b13] Pachos J. K. Introduction to Topological Quantum Computation Cambridge Univ. Press (2012).

[b14] Leek P. . Observation of Berry's phase in a solid state qubit. Science 318, 1889–1892 (2007). 18033851 10.1126/science.1149858

[b15] Abdumalikov A. A. . Experimental realization of non-Abelian geometric gates. Nature 496, 482–485 (2013). 23594739 10.1038/nature12010

[b16] Roushan P. . Observation of topological transitions in interacting quantum circuits. Nature 515, 241–244 (2014). 25391961 10.1038/nature13891

[b17] Bergmann K., Theuer H. & Shore B. W. Coherent population transfer among quantum states of atoms and molecules. Rev. Mod. Phys. 70, 1003–1025 (1998).

[b18] Vitanov N. V., Halfmann T., Shore B. W. & Bergmann K. Laser-induced population transfer by adiabatic passage techniques. Annu. Rev. Phys. Chem. 52, 763–809 (2001). 11326080 10.1146/annurev.physchem.52.1.763

[b19] Gaubatz U., Rudecki P., Schiemann S. & Bergmann K. Population transfer between molecular vibrational levels by stimulated Raman scattering with partially overlapping laser fields. a new concept and experimental results. J. Chem. Phys. 92, 5363–5376 (1990).

[b20] Du Y.-X., Liang Z.-T., Huang W., Yan H. & Zhu S.-L. Experimental observation of double coherent stimulated Raman adiabatic passages in three-level *λ* systems in a cold atomic ensemble. Phys. Rev. A 90, 023821 (2014).

[b21] Siewert J., Brandes T. & Falci G. Adiabatic passage with superconducting nanocircuits. Opt. Commun. 264, 435–440 (2006).

[b22] Siewert J., Brandes T. & Falci G. Advanced control with a Cooper-pair box: stimulated Raman adiabatic passage and Fock-state generation in a nanomechanical resonator. Phys. Rev. B 79, 024504 (2009).

[b23] Falci G. . Design of a lambda system for population transfer in superconducting nanocircuits. Phys. Rev. B 87, 214515 (2013).

[b24] Wei L. F., Johansson J. R., Cen L. X., S. Ashhab S. & Nori F. Controllable coherent population transfers in superconducting qubits for quantum computing. Phys. Rev. Lett. 100, 113601 (2008). 18517785 10.1103/PhysRevLett.100.113601

[b25] Koch J. . Charge insensitive qubit design derived from the Cooper pair box. Phys. Rev. A 76, 042319 (2007).

[b26] Wallraff A. . Strong coupling of a single photon to a superconducting qubit using circuit quantum electrodynamics. Nature 431, 162–167 (2004). 15356625 10.1038/nature02851

[b27] Bianchetti R. . Dynamics of dispersive single-qubit readout in circuit quantum electrodynamics. Phys. Rev. A 80, 043840 (2009).

[b28] Vasilev G. S., Kuhn A. & Vitanov N. V. Optimum pulse shapes for stimulated Raman adiabatic passage. Phys. Rev. A 80, 013417 (2009).

[b29] Torosov B. T. & Vitanov N. V. Composite stimulated Raman adiabatic passage. Phys. Rev. A 87, 043418 (2013).

[b30] Bianchetti R. . Control and tomography of a three level superconducting artificial atom. Phys. Rev. Lett. 105, 223601 (2010). 21231385 10.1103/PhysRevLett.105.223601

[b31] Vitanov N. V., Suominen K.-A. & Shore B. W. Creation of coherent atomic superpositions by fractional stimulated Raman adiabatic passage. J. Phys. B 32, 4535–4546 (1999).

[b32] Neeley M. . Emulation of a quantum spin with a superconducting phase qudit. Science 325, 722–725 (2009). 19661423 10.1126/science.1173440

[b33] Svetitsky E. . Hidden two-qubit dynamics of a four-level Josephson circuit. Nat. Commun. 5, 5617 (2014). 25421003 10.1038/ncomms6617

[b34] Møller D., Madsen L. B. & Mølmer K. Geometric phase gates based on stimulated Raman adiabatic passage in tripod systems. Phys. Rev. A 75, 062302 (2007).

[b35] Bal M., Ansari M. H., Orgiazzi J.-L., Lutchyn R. M. & Lupascu A. Dynamics of parametric fluctuations induced by quasiparticle tunneling in superconducting flux qubits. Phys. Rev. B 91, 195434 (2015).

[b36] Anisimov P. M., Dowling P., Jonathan & Sanders B. C. Objectively discerning Autler-Townes splitting from electromagnetically induced transparency. Phys. Rev. Lett. 107, 163604 (2011). 22107383 10.1103/PhysRevLett.107.163604

