# Supplementary material for: Stimulated Raman adiabatic passage in a three-level superconducting circuit
Source: Nat Commun. 2016 Feb 23;7:10628. doi: 10.1038/ncomms10628 (PMC4766393; doi:10.1038/ncomms10628)
Supplement: Supplementary Information — Supplementary Figures 1-5, Supplementary Notes 1-6 and Supplementary References [file ncomms10628-s1.pdf]

Supplementary Figures

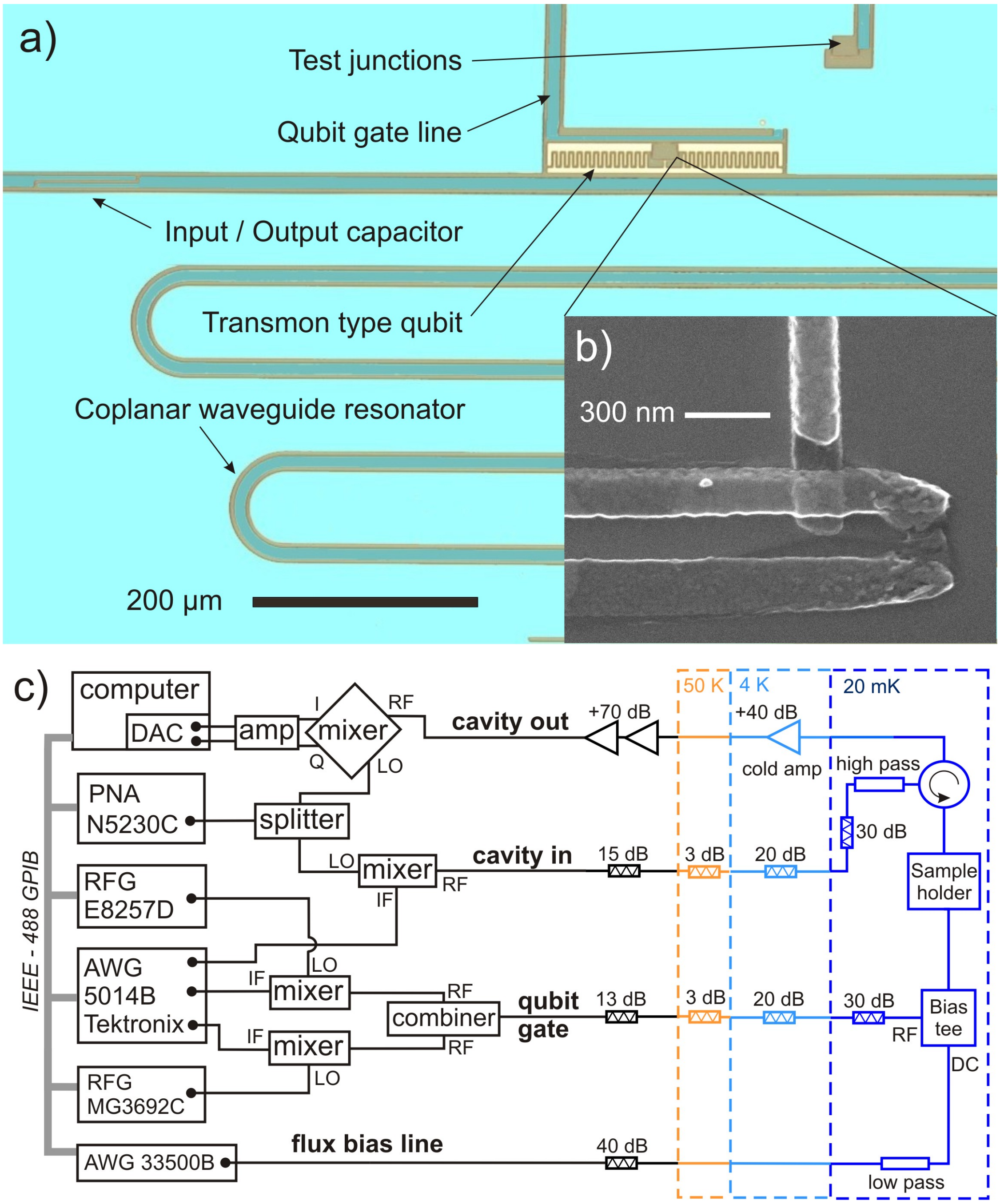

Supplementary Figure 1: Sample and measurement schematic. a) Optical microscope image (false colors) of the main elements of the sample. b) SEM image of one of the Josephson junctions in the transmon. c) Simplified schematic of the room temperature electronics, and the cryogenic setup and wiring used in the experiment.

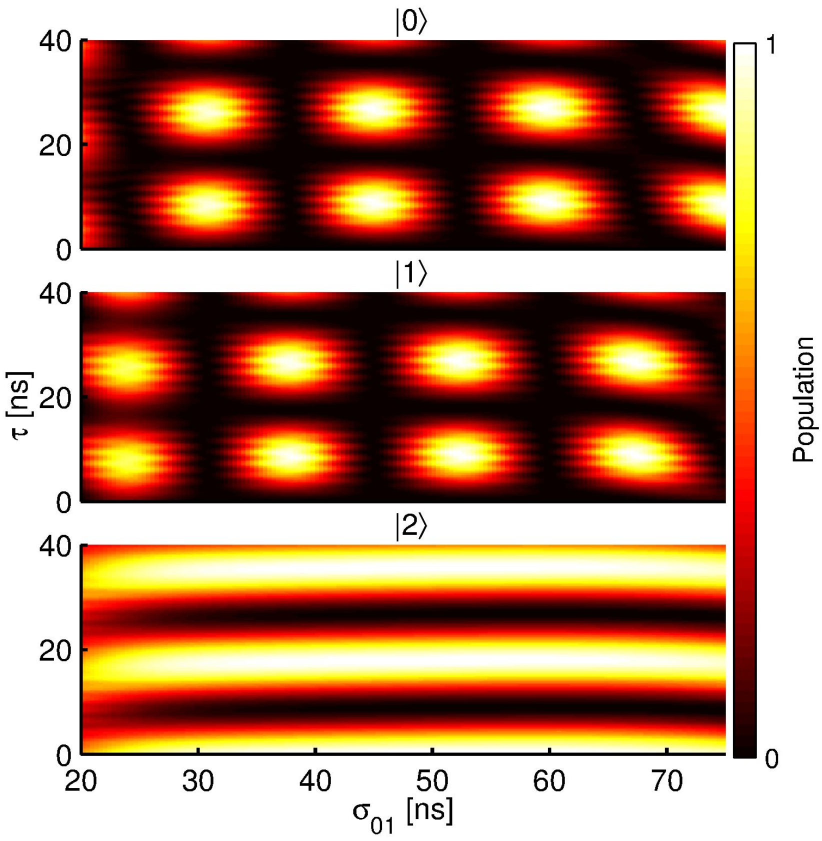

Supplementary Figure 2: State control with the hybrid sequence. The plot shows a simulation of the population on the three levels as a function of the width of the last $0-1$ pulse, σ_{01} (horizontal axis) and the length τ of the initial nonadiabatic pulse (vertical axis), in the dissipationless case. The separation between the STIRAP pulses was $t_s = -92$ ns, the same as in Fig. 4 from the main text.

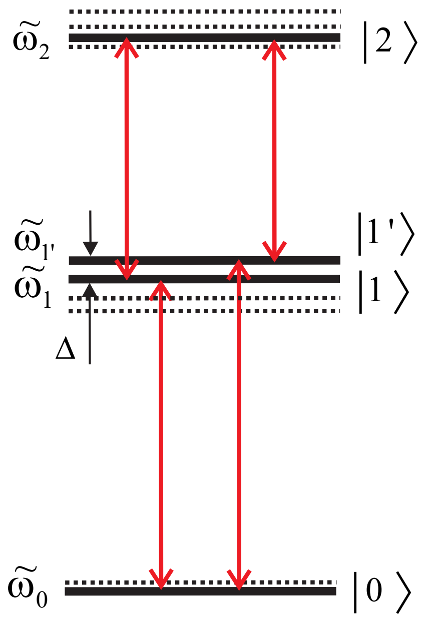

Supplementary Figure 3: Energy levels with a split first excited state. The diagram shows the energy levels with the first excited state split into $|1\rangle$ and $|1'\rangle$. The red arrows indicate the transitions driven by the applied fields. The dotted lines are other possible levels created after the thermal cycle.

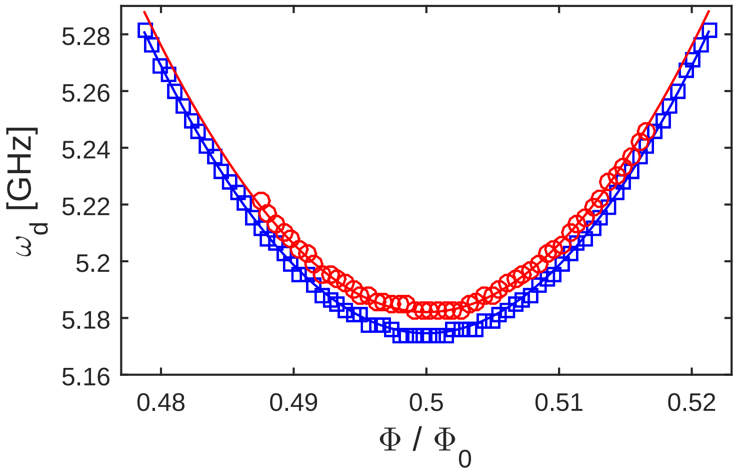

Supplementary Figure 4: The first transition frequency after thermal cycling. Plot of the first transition frequency of the transmon as a function of external magnetic field after the thermal cycling, showing the appearance of a split-line structure. Here the squares and the circles are the maximum values of the two peaks extracted from the experimental data. At larger values of the external flux the peak shifted upwards (red) becomes less visible. The continuous lines are fits with the expression of the first transition frequency of the transmon, see Ref. [1], with a shift $\delta E_J = h \times 27.5$ MHz in one of the junctions.

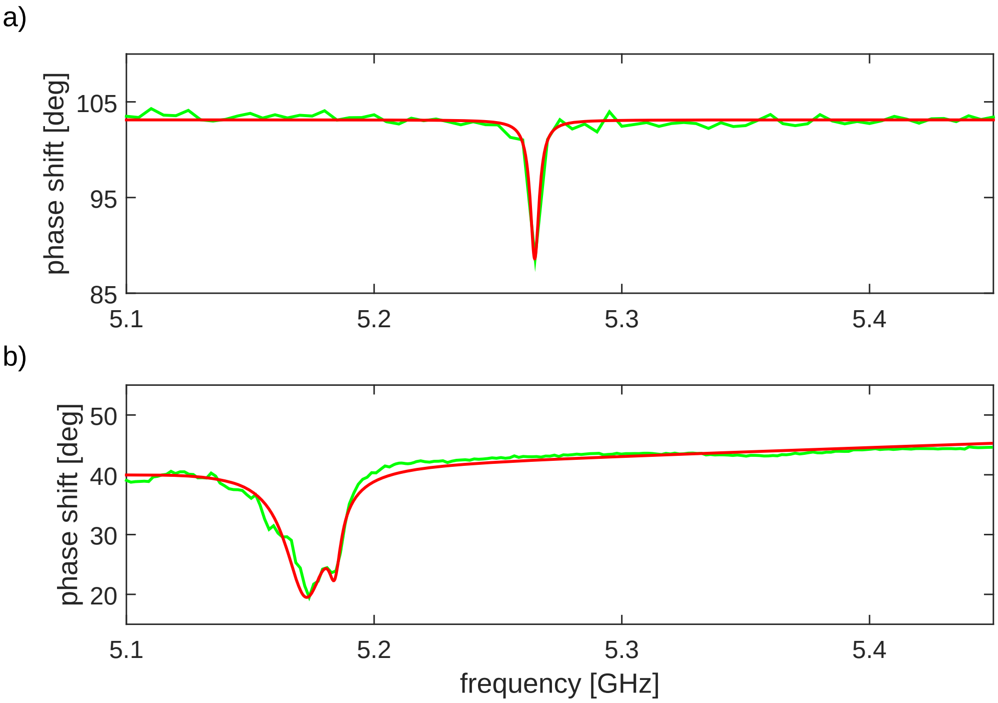

Supplementary Figure 5: Transmon spectrum before and after thermal cycling. We measure the phase shift of the cavity around the first transition frequency a) before and b) after the splitting at a flux point corresponding to the minimum of the transition frequency (see Supplementary Figure 4), where the transmon is the least sensitive to magnetic flux noise. The green lines represent experimental data, and the red lines are fits by using the least squares method a) with a single Lorentzian function and b) with a sum of two Lorentzian functions. See also Ref. [2] for more details.

Supplementary Note 1: Sample and measurement setup

The superconducting QED circuit [1] consists of a capacitively-shunted Cooper pair box (a transmon), with tunable energy level separation under the application of an external magnetic field. The qubit can be excited by applying a continuous wave or pulsed microwaves. The readout system is realized as a $\lambda/4$ coplanar waveguide cavity (resonator) which allows the quantum non-demolition measurement of the state of the transmon [3, 4]. A picture of the sample is shown in Supplementary Figure 1a),b), and a schematic of the measurement setup at room temperature and in the dilution refrigerator is presented in Supplementary Figure 1c).

The transmon is essentially an artificial atom with energy levels $\hbar\omega_j$ yielding transition frequencies denoted by $\omega_{j,j+1} = \omega_{j+1} - \omega_j$, and can be modelled as an anharmonic oscillator. With the standard notations in the field of superconducting devices, E_J denoting the Josephson energy of the qubit and E_C the total charge energy (including the shunt capacitor), the anharmonicity is $\hbar\omega_{12} - \hbar\omega_{01} \approx -E_\mathrm{C}$ in the asymptotic limit $E_\mathrm{J} \gg E_\mathrm{C}$. The usual way to manipulate the state of the circuit QED system is to apply microwave drive signals either to the gate of the transmon or to the coupled transmission line cavity. If the frequency of the drive $\omega^{(\Omega)}_{j,j+1}$ matches the transition frequency between two states of the system, the system goes through Rabi oscillations with frequency $\Omega_{j,j+1}$, resulting in transfer of population between the two states.

We model our system using the driven Jaynes-Cummings Hamiltonian describing a multilevel qubit coupled to a cavity (resonator) of frequency ω_res. A measurement tone of frequency ω_meas is coupled with strength ϵ_meas into the resonator from the input capacitor. The total Hamiltonian is then

$$\begin{aligned}\hat{H}_{JC} = &\sum_{j=0}^{N} \hbar\omega_j |j\rangle\langle j| + \hbar\omega_\mathrm{res}\hat{a}^\dagger\hat{a} + \sum_{j=0}^{N-1} \hbar g_{j,j+1} \left(|j+1\rangle\langle j|\hat{a} + \mathrm{h.c.}\right) \\ &+ \sum_{j=0}^{N-1} \frac{\hbar\Omega_{j,j+1}}{2} \left(|j+1\rangle\langle j| \mathrm{e}^{-i\omega^{(\Omega)}_{j,j+1}t} + \mathrm{h.c.}\right) + \hbar\epsilon_\mathrm{meas}\left(\hat{a}^\dagger \mathrm{e}^{-i\omega_\mathrm{meas}t} + \mathrm{h.c}\right),\end{aligned} \tag{1}$$

where $|j\rangle$ are the eigenstates of the N-level transmon corresponding to the eigenenergy $\hbar\omega_j$. Here we have applied the rotating wave approximation to the driving and the measurement fields, retaining only energy-conserving terms. We also only keep the coupling between consecutive energy levels with the corresponding near-resonant fields.

The single mode resonator, described by the annihilation (creation) operator $\hat{a}$ ($\hat{a}^\dagger$), couples to the qubit transition $j \to j+1$ with the Jaynes-Cummings coupling strength $g_{j,j+1}$. In the dispersive regime, when the qubit is detuned from the cavity and the number of photons in the resonator is not too large such that $4\langle \hat{a}^\dagger \hat{a}\rangle \left[g_{j,j+1}^2/(\omega_{j+1}-\omega_j-\omega_{\rm res})^2\right] \ll 1$, we can perform a generalized Schrieffer-Wolff transformation with displacement operator $\hat{D} = \exp\left[\hat{A}\right]$, where

$$\hat{A} = \sum_{j=0}^{N} \frac{g_{j,j+1}}{\omega_{j+1}-\omega_j-\omega_{\rm res}} \left(\hat{a}|j+1\rangle\langle j| - \text{h.c.}\right), \tag{2}$$

on the Hamiltonian in Eq. (1), resulting in $\hat{H} \to \hat{D}\hat{H}\hat{D}^\dagger$. Next, we expand

$$e^{\hat{A}}\hat{H}e^{-\hat{A}} = \hat{H} + \left[\hat{A},\hat{H}\right] + \frac{1}{2}\left[\hat{A},\left[\hat{A},\hat{H}\right]\right] ..., \tag{3}$$

with $g_{j,j+1}/(\omega_{j+1}-\omega_j-\omega_{\rm res})$ as a small parameter, and we retain terms up to second order. The Jaynes-Cummings interaction between the qubit and the resonator cancels exactly and we are left with

$$\begin{aligned}\hat{H}_{\rm disp-JC} =& \sum_{j=1}^{N} \hbar\left(\omega_j + \chi_{j-1,j}\right)|j\rangle\langle j| - \hbar\chi_{0,1}\hat{a}^\dagger\hat{a}|0\rangle\langle 0| \\ &+ \hbar\omega_{\rm res}\hat{a}^\dagger\hat{a} + \sum_{j=1}^{N}\hbar\left(\chi_{j-1,j}-\chi_{j,j+1}\right)\hat{a}^\dagger\hat{a}|j\rangle\langle j| \\ &+ \sum_{j=0}^{N-1}\frac{\hbar\Omega_{j,j+1}}{2}\left(|j+1\rangle\langle j|\mathrm{e}^{-i\omega_{j,j+1}^{(\Omega)}t} + \text{h.c.}\right) + \hbar\epsilon_{\rm meas}\left(\hat{a}^\dagger\mathrm{e}^{-i\omega_{\rm meas}t} + \text{h.c}\right).\end{aligned} \tag{4}$$

Here the dispersive shifts $\chi_{j,j+1}$ are defined as

$$\chi_{j,j+1} = \frac{g_{j,j+1}^2}{\omega_{j+1}-\omega_j-\omega_{\rm res}}. \tag{5}$$

In obtaining Eq. (4), several terms have been neglected. A two-photon Hamiltonian resulting from the Jaynes-Cummings interaction couples states separated by two ladder indices,

$$\sum_{j=0}^{N-2}\frac{(2\omega_{j+1}-\omega_j-\omega_{j+2})\hbar g_{j,j+1}g_{j+1,j+2}}{2(\omega_{j+2}-\omega_{j+1}-\omega_{\rm res})(\omega_{j+1}-\omega_j-\omega_{\rm res})}\hat{a}^2|j+2\rangle\langle j| + \text{h.c.}. \tag{6}$$

Then there are terms resulting from the commutator of $\hat{A}$ with the drive,

$$\begin{aligned}&\sum_j \frac{g_{j,j+1}}{\omega_{j+1}-\omega_j-\omega_{\rm res}} a\left[\frac{\hbar\Omega_{j-1,j}}{2}e^{-i\omega_{j-1,j}^{(\Omega)}t}|j+1\rangle\langle j-1|\right. \\ &\left.-\frac{\hbar\Omega_{j+1,j+2}}{2}e^{-i\omega_{j+1,j+2}^{(\Omega)}t}|j+2\rangle\langle j| + \frac{\hbar\Omega_{j,j+1}}{2}e^{i\omega_{j,j+1}^{(\Omega)}t}\left(|j+1\rangle\langle j+1| - |j\rangle\langle j|\right)\right],\end{aligned} \tag{7}$$

and with the measurement pulse

$$\hbar\epsilon_{\rm meas}\sum_j \frac{g_{j,j+1}}{\omega_{j+1}-\omega_j-\omega_{\rm res}}\left[|j+1\rangle\langle j|e^{-i\omega_{\rm meas}t}+{\rm h.c.}\right]. \tag{8}$$

Several arguments can be invoked to neglect these terms: some are second-order detuned processes such as Eq. (6), and the rest would produce only first-order corrections to the dominant driving term. Moreover, when the qubit is driven, the resonator is not populated, and therefore the relevant terms left in Eq. (7) contribute only as a renormalization of the decoherence of the second excited state.

Next, we set the ground state as a reference for the energy levels by subtracting a quantity $\hbar\omega_0\sum_{j=1}^N|j\rangle\langle j|$ from the resulting Hamiltonian. Further, we move into a multiple-rotating frame defined by the transformation

$$\hat{U}(t)=|0\rangle\langle 0|+\sum_{j=1}^N \exp\left[i\sum_{k=0}^{j-1}\omega_{k,k+1}^{(\Omega)}t\right]|j\rangle\langle j|, \tag{9}$$

which transforms the Hamiltonian as $\hat{H}\rightarrow\hat{U}\hat{H}\hat{U}^\dagger+i\hbar(d\hat{U}/dt)\hat{U}^\dagger$. We also perform a rotation with respect to the cavity at the measurement frequency, $\hat{U}_{\rm meas}=\exp\left[i\omega_{\rm meas}\hat{a}^\dagger\hat{a}t\right]$ resulting in a similar transformation $\hat{H}\rightarrow\hat{U}_{\rm meas}\hat{H}\hat{U}^\dagger_{\rm meas}+i\hbar(d\hat{U}_{\rm meas}/dt)\hat{U}^\dagger_{\rm meas}$. Finally, this yields

$$\begin{aligned}\hat{H}_{\rm disp-JC}=&\sum_{j=1}^N\hbar\left(\omega_j-\omega_0+\chi_{j-1,j}-\sum_{k=0}^{j-1}\omega^{(\Omega)}_{k,k+1}\right)|j\rangle\langle j|-\hbar\chi_{0,1}\hat{a}^\dagger\hat{a}|0\rangle\langle 0|\\&+\hbar\left(\omega_{\rm res}-\omega_{\rm meas}\right)\hat{a}^\dagger\hat{a}+\sum_{j=1}^N\hbar\left(\chi_{j-1,j}-\chi_{j,j+1}\right)\hat{a}^\dagger\hat{a}|j\rangle\langle j|\\&+\sum_{j=0}^{N-1}\frac{\hbar\Omega_{j,j+1}}{2}\left(|j+1\rangle\langle j|+{\rm h.c.}\right)+\hbar\epsilon_{\rm meas}\left(\hat{a}+\hat{a}^\dagger\right).\end{aligned} \tag{10}$$

The excitation of the qubit to the energy level $j\geq 1$ results in a measurable shift of the resonator transition frequency by $\chi_{j-1,j}+\chi_{0,1}-\chi_{j,j+1}$, thus enabling a quantum non-demolition measurement of the transmon state. This is realized by monitoring the response of the cavity near the resonance under the application of a measurement pulse. Before the measurement field is applied, that is during the STIRAP pulses, the number of photons in the cavity - and therefore the ac-Stark shift on the transmon - is negligible. In this case the only effect of the resonator on the qubit is that its vacuum fluctuations produce a small Lamb-shifted renormalization of the energy levels, $\tilde{\omega}_j=\omega_j+\chi_{j-1,j}$ for $j\geq 1$ and $\tilde{\omega}_0=\omega_0$, with corresponding

transition frequencies $\tilde{\omega}_{j,j+1} = \tilde{\omega}_{j+1} - \tilde{\omega}_j$. Let us introduce the detunings as

$$\delta_{j,j+1} = \omega_{j+1} + \chi_{j,j+1} - \omega_j - \chi_{j-1,j} - \omega_{j,j+1}^{(\Omega)}$$

for $j \geq 1$ and $\delta_{01} = \omega_1 + \chi_{01} - \omega_0 - \omega_{01}^{(\Omega)}$ for the first transition.

Next we focus on the STIRAP by considering only the three lowest eigenstates of the transmon, i.e. the states $|0\rangle, |1\rangle$, and $|2\rangle$. This eventually leads to a very simple three-level form for the system Hamiltonian

$$\hat{H}(t) = \frac{\hbar}{2} \begin{bmatrix} 0 & \Omega_{01}(t) & 0 \\ \Omega_{01}(t) & 2\delta_{01} & \Omega_{12}(t) \\ 0 & \Omega_{12}(t) & 2(\delta_{01} + \delta_{12}) \end{bmatrix}. \quad (11)$$

To simplify the notation, in the expression above as well as in the main paper we have eliminated the comma between subscript indices whenever this does not lead to confusion, for example we write $g_{0,1} \equiv g_{01}$, $\omega_{0,1} \equiv \omega_{01}$, $\Omega_{0,1} \equiv \Omega_{01}$.

Several sources of noise affect the transmon, as discussed in detail in Ref. [1]. The main advantage of the transmon with respect to the previously used charge qubit, from which it is derived, is the insensitivity to charge noise. The flux noise can be minimized by operating the device near the so-called flux sweet point, where the first-order derivatives of the transition frequency with respect to external flux cancel. For relaxation, important decay channels are the measuring resonator, the line used for magnetic flux biasing, and spurious electromagnetic modes of the sample holder, ground planes, *etc.*. To simulate the decoherence, we use the master equation for the density matrix in Lindblad form, see Methods Eqs. (7,8). Thus we assume that the Markovian approximation is valid, that is, that the characteristic decay time of the autocorrelation function of the environment is much smaller than the inverse of the decoherence rates $\Gamma_{j,k}^{-1}, (\Gamma_{j,k}^{\varphi})^{-1}$, with $j > k$ and $j, k \in \{0, 1, 2\}$. For relaxation processes, this approximation is well satisfied due to the fact that modes such as those of the flux bias line are dissipative, and resonances such as those from the measuring coplanar waveguide cavity and those of the sample holder are far off detuned from the qubit. We assume that this holds as well for the dephasing processes. For our sample, the decoherence is anyway dominated by relaxation processes, therefore this approximation is not critical. This is supported also by the fact that, when extracting the decoherence times, we find that the exponential decay predicted by the Lindblad form gives a very good matching to the data.

Supplementary Note 2: Derivation of the global adiabaticity condition

Adiabatic processes should satisfy $|\langle\pm|(d/dt)|\mathrm{D}\rangle| \ll |\omega_\pm - \omega_\mathrm{D}|$, which, when employing the expressions of the eigenvalues presented in Methods, leads to

$$\left|\frac{\dot{\Omega}_{01}(t)\Omega_{12}(t) - \Omega_{01}(t)\dot{\Omega}_{12}(t)}{[\Omega_{01}(t)^2 + \Omega_{12}(t)^2]^{3/2}}\right| \ll 1, \tag{12}$$

for $\delta_{01} = 0$ and $\sin\Phi = 1$.

For Gaussian pulse shapes with equal amplitudes

$$\Omega_{01}(t) = \Omega \exp\left[-\frac{t^2}{2\sigma^2}\right], \tag{13}$$

$$\Omega_{12}(t) = \Omega \exp\left[-\frac{(t-t_s)^2}{2\sigma^2}\right], \tag{14}$$

we can find a global adiabatic condition by integrating Eq. (12) over time

$$\frac{\pi}{2} \ll \int_{-\infty}^{\infty} \sqrt{\Omega_{01}(t)^2 + \Omega_{12}(t)^2}\,\mathrm{d}t. \tag{15}$$

The remaining integral above does not have an analytical solution. However, it can be shown that it is an increasing function of t_s; moreover, the values at $t_s = 0$ and $t_s = \infty$ can be calculated analytically, and they are $2\sqrt{\pi}\sigma\Omega$ and $2\sqrt{2\pi}\sigma\Omega$, respectively. Thus, we have to select the minimum value $2\sqrt{\pi}\sigma\Omega$ to obtain the constraint, and we get

$$\frac{4}{\sqrt{\pi}}\sigma\Omega \gg 1. \tag{16}$$

Supplementary Note 3: Calibration and measurement protocol

We first determine the transition frequencies of the transmon by measuring the Rabi frequency of a driven transition as a function of the drive frequency. This gives $\widetilde{\omega}_{01}/2\pi = 5.27$ GHz, $\widetilde{\omega}_{12}/2\pi = 4.82$ GHz.

The used measurement setup is shown in Supplementary Figure 1. To characterize the STIRAP process we determine the state of the three-level transmon by probing the coupled transmission line cavity with a measurement signal. Due to the dispersive shift in the cavity resonance frequency, the response of the cavity to the probing microwave signal is different depending on the state of the transmon. To measure the cavity response we apply a homodyne detection scheme, where the incoming microwave signal is downconverted to DC using an IQ-mixer. The resulting in-phase and quadrature signals are given by

$$\begin{aligned} \langle \hat{I}(\tau) \rangle &= -\eta \langle \hat{a} + \hat{a}^\dagger \rangle, \\ \langle \hat{Q}(\tau) \rangle &= i\eta \langle \hat{a} - \hat{a}^\dagger \rangle, \end{aligned} \tag{17}$$

where η is a factor describing the losses of the conversion and the other constants. By preparing the transmon in the states $|0\rangle$, $|1\rangle$, and $|2\rangle$ and then measuring the corresponding cavity response, we can associate the response of each of the states with a variable $r_j(\tau) = \{\langle \hat{I}_j(\tau) \rangle, \langle \hat{Q}_j(\tau) \rangle\}$, and then describe the cavity response of every other state as a linear combination of $r_j(\tau)$

$$r_{\rm meas}(t, \tau) = \sum_{j=0,1,2} p_j(t) r_j(\tau). \tag{18}$$

The measured signal is therefore a linear combination of the responses of each state, weighted by the population $p_j(t) = \mathrm{Tr}[\rho(t)|j\rangle\langle j|]$ of the state, $\sum_{j=0,1,2} p_j(t) = 1$ and $0 \leq p_j(t) \leq 1$ [5]. This can be understood by introducing the operator corresponding to $r_{\rm meas}(t, \tau)$, $\hat{r}_{\rm meas}(\tau) = \sum_{j=0,1,2} r_j(\tau)|j\rangle\langle j|$ and thus obtaining Eq. (18) as $r_{\rm meas}(t, \tau) = \mathrm{Tr}[\rho(t)\hat{r}_{\rm meas}(\tau)]$.

For a given trace $r_{\rm meas}(t, \tau)$ in the $I - Q$ plane, we determine p_0, p_1, and p_2 by applying the least square fit method to invert Eq. (18). To implement the least squares fit method we employ the Levenberg-Marquardt algorithm, using the measurement response data up to $\tau = 1500$ ns. As pre-processing, the data is multiplied with an exponential weighting function $\exp[-\tau/w]$, with $w = 700$ ns. This allows us to use predominantly the data from the beginning of the response curves, where the effect of decoherence on the distinguishability of the states is minimal.

In Fig. 1c) in the main text we present the $\langle \hat{I}(\tau) \rangle$ traces corresponding to the system in the ground state (blue), first excited state (green), and second excited state (red), together with the trace for the density matrix at a time $t = 450$ ns (cyan) during the STIRAP protocol. The calibration trace of the first excited state is obtained by applying a resonant π pulse to the $|0\rangle \rightarrow |1\rangle$ transition, while the calibration trace of the second excited state is obtained by first populating the first excited state with a π pulse, and then applying another π pulse in resonance with the $|1\rangle \rightarrow |2\rangle$ transition. This allows us to infer the density matrix of the system, given a set of measured $\langle \hat{I}(\tau) \rangle, \langle \hat{Q}(\tau) \rangle$ traces. The main source of error in this procedure is produced by decoherence. The decay from the upper states during the calibration pulses results in a small inaccuracy, which produces a small overestimation of the populations in the tomography. We minimize this error as postprocessing by including the effect of decoherence in the calibration.

Supplementary Note 4: Hybrid sequence

In Fig. 4 in the main text we present the results of applying a nonadiabatic (hard) pulse on the $0-1$ transition before the STIRAP sequence. This creates a superposition of the ground state and the first excited state as the initial state for STIRAP. As a result, the population on state $|0\rangle$ that can be transferred to state $|2\rangle$ by STIRAP is reduced compared to the situation without the Rabi pulse. Also, because now the state $|1\rangle$ is populated, the STIRAP pulses will produce Rabi oscillations between all the states.

From Fig. 4 we can see that the population on the state $|2\rangle$ stabilizes to a non-oscillatory value immediately after the overlap between the adiabatic pulses, exactly like in the standard STIRAP sequence, while the population of states $|0\rangle$ and $|1\rangle$ continues to oscillate until the end of the last adiabatic $0-1$ pulse. After the extinction of the $1-2$ pulse, the period of these oscillations is determined only by the remaining $0-1$ pulse at the end of the sequence, and, as can be seen by inspecting the figure along the vertical axis, this period is independent of the pulse length of the nonadiabatic pulse. In the case of state $|2\rangle$, its final population is determined only by the length of the initial remaining population on the state $|0\rangle$ after the nonandiabatic pulse, and it is independent of the parameters of the STIRAP sequence. To show this, in Supplementary Figure 2 we present the results of a simulation of the population of all the states at the end of a hybrid sequence as a function of the width σ_{01} of the Gaussian $0-1$ pulse (horizontal axis) and for a variable length of the nonadiabatic pulse (vertical axis). For simplicity we consider here the dissipationless case. One can clearly see the features mentioned above, with only small modifications at small σ_{01}, where the adiabatic condition is not well satisfied.

These hybrid sequences therefore enable the preparation of three-level states with populations determined by independent pulses. This can be used as a novel tool in quantum control of qutrits. For example, in our experiment, for nonadiabatic pulse lenght $\tau = 25$ ns we have prepared near the end of the hybrid sequence at $t = 283$ ns a state with populations $p_0 = 0.37$, $p_1 = 0.33$, $p_2 = 0.30$. This is not exactly a pure state because of decoherence, but nevertheless it is close to an equal-superposition state between the three levels.

Supplementary Note 5: STIRAP as a route to holonomic quantum computing

The phases of the driving fields, which we ignored so far, can lead to interesting new ways of qubit manipulation through the accumulation of geometric phases. For completeness, here we briefly discuss this situation. We show that the Hamiltonian in this case is identical to that used in proposals for holonomic quantum gates, and we calculate the Berry phase arising in the STIRAP process [6]. If the phase factors are retained in the driving fields, we obtain the following three-level form for the system Hamiltonian

$$\hat{H}(t) = \frac{\hbar}{2}\begin{bmatrix} 0 & \Omega_{01}^{*} & 0 \\ \Omega_{01} & 2\delta_{01} & \Omega_{12} \\ 0 & \Omega_{12}^{*} & 2(\delta_{01}+\delta_{12}) \end{bmatrix}. \tag{19}$$

Let us denote by φ the phase difference between the driving fields, therefore we can take $\Omega_{01} = |\Omega_{01}|$, $\Omega_{12} = |\Omega_{01}|\exp(-i\varphi)$. At two-photon resonance $\delta_{01} + \delta_{12} = 0$, the eigenvalues/eigenvectors problem at a time t has the solution $\omega_{+} = \delta_{01} + \sqrt{\delta_{01}^2 + |\Omega_{01}|^2 + |\Omega_{12}|^2}$, $\omega_{-} = \delta_{01} - \sqrt{\delta_{01}^2 + |\Omega_{01}|^2 + |\Omega_{12}|^2}$, and $\omega_{\mathrm{D}} = 0$, with corresponding eigenvectors

$$\begin{aligned} |+\rangle &= \sin\Phi|B\rangle + \cos\Phi|1\rangle, \\ |-\rangle &= \cos\Phi|B\rangle - \sin\Phi|1\rangle, \\ |\mathrm{D}\rangle &= \cos\Theta|0\rangle - \sin\Theta e^{i\varphi}|2\rangle, \end{aligned} \tag{20}$$

where the bright state is $|B\rangle = \sin\Theta|0\rangle + \cos\Theta e^{i\varphi}|2\rangle$. The angle Θ is defined by $\tan\Theta = |\Omega_{01}(t)|/|\Omega_{12}(t)|$ and parameterizes the rotation in the $\{|0\rangle, |2\rangle\}$ subspace, while Φ is defined in the same way, as an angle of a right triangle with vertices $\sqrt{(|\Omega_{01}|^2 + |\Omega_{12}|^2)/2}$ and $\sqrt{(|\Omega_{01}|^2 + |\Omega_{12}|^2 + \delta_{01}^2)/2} + \delta_{01}/\sqrt{2}$.

The Berry phase accumulated during a STIRAP sequence can be calculated from the standard Berry's connection formula

$$\gamma_{\mathrm{Berry}} = i\int_{R_i}^{R_f} \langle \mathrm{D}|\nabla_{\mathbf{R}}|\mathrm{D}\rangle d\mathrm{R}, \tag{21}$$

where the integral is taken along a contour in the (Θ, φ) space,

$$\mathbf{R} = \begin{pmatrix} \Theta \\ \varphi \end{pmatrix}. \tag{22}$$

For a general trajectory starting at an initial time t_i, this yields at time t [6]

$$\gamma_{\text{Berry}}(t) = -\int_{\varphi(t_i)}^{\varphi(t)} \sin^2 \Theta d\varphi. \tag{23}$$

Because the energy eigenvalue of the dark state is zero, from the time t_i to time t the system would only pick up a geometrical phase,

$$|\text{D}(t_i)\rangle \rightarrow e^{i\gamma_{\text{Berry}}(t)}|\text{D}(t)\rangle. \tag{24}$$

After a full STIRAP process the state then changes as

$$|0\rangle \rightarrow -e^{i\gamma_{\text{Berry}}(t_f)+i\varphi(t_f)}|2\rangle. \tag{25}$$

In holonomic quantum computing, the geometric phase γ_{Berry} is used to construct single-qubit phase gates.

Supplementary Note 6: STIRAP with a split intermediate state

We analyze here in more detail the results presented in Fig. 6, with the aim of providing an understanding of the physical mechanism responsible for this structure. Before embarking into evaluating possible microscopic models, a few important observations are in place. Firstly, if we examine the pattern along the single-photon detuning axis (vertical axis) one notices that we have two distinct half-diamond regions where the transfer is maximum. This means that there are two single-photon resonances instead of one, corresponding to a splitting of the first excited state of the transmon. Importantly, this structure cannot be understood simply as the average of two single-diamond patterns shifted along the vertical axis - this would only result in a broader diamond, without explaining the extinction of transfer in the middle of the structure. Thus, the system has to be treated fully quantum-mechanically, allowing for destructive interference effects. Secondly, at the power levels used in Fig. 6 we see no splitting along the two-photon transition in the pattern, implying that the state $|2\rangle$ remains unmodified with respect to $|0\rangle$. This shows that a minimal model, without splitting in the $|0\rangle \rightarrow |2\rangle$ transition, might be able to explain the pattern.

To make progress, it is useful to examine additional experimental data. The strategy that we follow has been outlined in Ref. [7]: although the system might have a complicated multilevel structure, one can examine the absorption spectra, select some of the energy levels, and consider the others as part of the dissipation. In practice, the choice is made easier by the fact that different transitions couple with different strengths to the microwave field, and some are seen only at relatively high microwave powers. In the two-tone spectroscopy of the first excited state we can observe the effect of the thermal cycling, see Supplementary Figure 4 and Supplementary Figure 5. Clearly the device has somewhat degraded, as the narrow, well-defined absorption spectrum before the warm-up has broadened and has acquired additional structure. For Supplementary Figure 5 we follow the procedure in Ref. [2] and we extract the values of the transition frequencies by fitting each peak with a Lorentzian function. We mention that the width of these peaks include in general the effects of power broadening: the powers used to measure the spectra presented in Supplementary Figure 5 a) and b) were different, thus these widths cannot be compared directly. From the spectral structure in Supplementary Figure 5 we retain only the two more prominent absorbtion dips, separated by $\Delta/(2\pi) \approx 11.5$ MHz. We have also checked that these peaks remain robust over a larger

range of microwave powers.

To avoid introducing too many notations, we will refer to these two first-excited states as $|1\rangle$ and $|1'\rangle$, see Supplementary Figure 3. The detunings with respect to the state $|1'\rangle$ are defined as $\delta_{01'} = \tilde{\omega}_{01'} - \omega_{01}^{(\Omega)} = \Delta + \delta_{01}$ and $\delta_{1'2} = \tilde{\omega}_{1'2} - \omega_{12}^{(\Omega)} = -\Delta + \delta_{12}$. In this way, the detunings with respect to state $|1\rangle$ are sufficient to label Fig. 6 of the main text, since the state $|1'\rangle$ is only displaced by Δ.

The Hamiltonian then reads

$$\hat{H}_{\text{transmon}} = \sum_{j=0}^{2} \hbar\tilde{\omega}_j |j\rangle\langle j| + \hbar\tilde{\omega}_{1'}|1'\rangle\langle 1'|. \tag{26}$$

Note that the separation Δ extracted from spectroscopy corresponds to a split of the same magnitude Δ along the single-photon axis in Fig. 6. Indeed, using our definitions one can check that at two-photon resonance $\delta_{01} = -\delta_{12} = \tilde{\omega}_{01} - \omega_{01}^{(\Omega)} = -(\tilde{\omega}_{12} - \omega_{12}^{(\Omega)})$.

Now, because we can see both levels $|1\rangle$ and $|1'\rangle$ in the spectroscopy, it means that the microwave field couples to both of the transitions $|0\rangle \to |1\rangle$ and $|0\rangle \to |1'\rangle$, therefore we can write

$$\begin{aligned}\hat{H}_{\text{drive}} &= \frac{\hbar\Omega_{01}}{2} e^{-i\omega_{01}^{(\Omega)}t}|1\rangle\langle 0| + \frac{\hbar\Omega_{01'}}{2} e^{-i\omega_{01}^{(\Omega)}t}|1'\rangle\langle 0| \\ &\quad + \frac{\hbar\Omega_{12}}{2} e^{-i\omega_{12}^{(\Omega)}t}|2\rangle\langle 1| + \frac{\hbar\Omega_{1'2}}{2} e^{-i\omega_{12}^{(\Omega)}t}|2\rangle\langle 1'| \\ &\quad + \text{h.c.},\end{aligned}$$

In the simulations, we use the Hamiltonian $\hat{H} = \hat{H}_{\text{transmon}} + \hat{H}_{\text{drive}}$ in the master equation. The Hamiltonian can also be transformed into a rotating frame, by following the same procedure as for Eq. (11). Explicitly, the operator $\hat{U}(t)$ is now

$$\hat{U}(t) = |0\rangle\langle 0| + e^{i\omega_{01}^{(\Omega)}t}\left(|1\rangle\langle 1| + |1'\rangle\langle 1'|\right) + e^{i(\omega_{01}^{(\Omega)} + \omega_{12}^{(\Omega)})t}|2\rangle\langle 2|, \tag{27}$$

and we obtain

$$\hat{H}(t) = \frac{\hbar}{2}\begin{bmatrix} 0 & \Omega_{01}(t) & \Omega_{01'}(t) & 0 \\ \Omega_{01}(t) & 2\delta_{01} & 0 & \Omega_{12}(t) \\ \Omega_{01'}(t) & 0 & 2\Delta + 2\delta_{01} & \Omega_{1'2}(t) \\ 0 & \Omega_{12}(t) & \Omega_{1'2}(t) & 2(\delta_{01} + \delta_{12}) \end{bmatrix}. \tag{28}$$

In this representation, the diagonal matrix element $|1'\rangle\langle 1'|$ is $2\delta_{01'} = 2\Delta + 2\delta_{01}$, while the diagonal matrix element $|2\rangle\langle 2\rangle$ can be equally well written in terms of the state $1'$, since $\delta_{01} + \delta_{12} = \delta_{01'} + \delta_{1'2}$ represents the two-photon detuning between state $|0\rangle$ and $|2\rangle$.

We now discuss possible microscopic mechanisms that would lead to this splitting. In the case of the flux qubit, it was proposed that the process is related to nonequilibrium quasiparticles, which would shift the frequency of the qubit between two values. For the transmon, the resulting frequency shifts can be calculated using the formula for the peak-to-peak dispersion from Ref. [1],

$$\epsilon_m \approx (-1)^m E_{\mathrm{C}} \frac{2^{2m+5}}{m!} \sqrt{\frac{2}{\pi}} \left(\frac{E_{\mathrm{J}}}{E_{\mathrm{C}}}\right)^{\frac{m}{2}+\frac{3}{4}} e^{-\left(\frac{8E_{\mathrm{J}}}{E_{\mathrm{C}}}\right)^{\frac{1}{2}}}. \tag{29}$$

However, it is not necessary that this mechanism is at work in a given device: this depends on many technical details, ranging from fabrication details to filtering. It is also not clear why it would be activated by a thermal cycling of the sample. However, we can calculate for our case the maximum frequency shifts that it would induce. Due to the exponent in the expression Eq. (29), large values of ϵ_m are obtained for small values of the Josephson energy E_{J} of the device. Following the standard convention in the field set by Ref. [1], E_J includes the effects of junction asymmetry and flux bias, and, after the thermal cycle, it reaches a minimum value of $E_{\mathrm{J}} = h \times 10.24$ GHz when $\tilde{\omega}_{01}$ reaches its minimum. Using $E_{\mathrm{C}}/h = 0.38$ GHz, which is determined independently from the geometric capacitance of the circuit, this yields $E_{\mathrm{J}}/E_{\mathrm{C}} = 27.2$. This predicts a shift of 1.6 MHz in the frequency of the first transition $|0\rangle \rightarrow |1\rangle$, which is insufficient to explain our splitting. This number is in full agreement with the result of experiments where measurement aimed at detecting these dispersive effects have been performed, for example in the work of Ref. [8] where shifts of the order of at most 1 MHz have been measured with device with E_J/E_{C} ratio of 25. For the second transition $|1\rangle \rightarrow |2\rangle$ the shift predicted by Eq. (29) is 47.7 MHz, which we do not observe in the spectroscopy of either this transition or of the two-photon transition from the ground state to the second excited state. These numbers are obviously quite different from the splitting we see in the experiment, therefore we conclude that this mechanism is ruled out.

Another mechanism we could consider is the resonant interaction with a two-level systems with exactly the same energy level separation as the $|0\rangle \rightarrow |1\rangle$ transition. For example, an electrical dipole-dipole interaction with strength $\hbar\Delta/2$ can be regarded as an $X-X$ coupling between the transmon and the resonant dipole, which would yield a splitting of Δ in the first transition. Indeed, if we denote the states of the two-level system as $|g\rangle$ and $e\rangle$, we can

write an interaction Hamiltonian

$$\frac{\hbar\Delta}{2}\left(|0e\rangle\langle 1g| + |1g\rangle\langle 0e|\right), \tag{30}$$

where we used the rotating wave approximation. Then, the subspace spanned by $|0e\rangle$ and $|1g\rangle$ is decoupled from the rest of the 4×4 Hilbert space; two eigenvalues of the interaction Hamiltonian, namely $\frac{1}{\sqrt{2}}\left(|1g\rangle \pm |0e\rangle\right)$ are formed, with eigenvalues $\pm\hbar\Delta/2$ respectively. Indeed, this type of splitting occurs very often in qubits with a large junction area, such as the phase qubits, where it was first observed and later studied in detail [9, 10], and recently it has been shown that these fluctuators can be tuned by an externally applied strain [11]. However, this model implies also that, when varying the energy level separation between the state $|0\rangle$ and $|1\rangle$, an avoided crossing is formed near the region where the transmon transition frequency is nearly resonant with that of the two-level system. This has been indeed observed in many experiments with phase qubits. To evaluate this possibility, we investigated the spectrum of the device as a function of magnetic field, see Supplementary Figure 4. We do not see evidence of an avoided crossing: rather, it looks as if the transition frequency is simply shifted between two values for a large range of magnetic fields applied.

This suggests that the nature of the coupling is parametric rather than of $X - X$ type and on-resonance. This type of coupling has not been explored much in the literature, with the notable exception of the theoretical investigation in Ref. [7] and the experiment in Ref. [12]. A first observation is that the spectral data suggest that the additional energy levels are associated with some modifications in the barrier. This is consistent with the previously reported formation of defects in a flux qubit that was warmed up and cooled down again [13]. As one can see from Supplementary Figure 4, the thermal cycle has produced not only level splitting but also a frequency shift in the energy level by approx. 90 MHz, which we observe also in the second transition. Since E_C is fixed by geometry, it is natural to suspect that something has happened in the barrier of the junctions which reduced E_J at the flux insensitive point and brought the oxide layer in an unstable state. In Supplementary Figure 4 the fit of the expression for the lower (blue colour) transition frequency of the transmon corresponds to Josephson energies of $h \times 27.25$ GHz and $h \times 17.01$ GHz for the two junctions of the SQUIDs comprising the transmon, see Ref. [1]. Interestingly, these fabrication parameters obtained from similar measurements before the warmup were $h \times 5.99$ GHz and $h \times 16.47$ GHz, which suggests that the change could have occurred only in one

junction and consisted in the opening of additional conduction channels. The red line in Supplementary Figure 4 is obtained by shifting the Josephson energy of the $h \times 27.25$ GHz junction up by $h \times 27.5$ MHz. This choice is of course not unique, and one could fit the data by lowering the Josephson energy of the $h \times 17.01$ GHz junction by approximately the same amount or by modifying the Josephson energies of both junctions. At the minimum of the transition frequency (flux insensitive point) this yields a line separation of ≈ 11.5 MHz.

The formation of fluctuating defects has been studied for a long time in glasses and amorphous solids at low temperatures, where they modify the temperature-dependence of the specific heat and thermal conductivity [14, 15]. In the case of AlO_x the mechanism is the delocalization of the oxygen atoms from the non-crystalline structure. This results in the formation of a non-harmonic system with a quasi-degenerate ground state, with one oxygen atom sitting in a double well potential and jumps between the two positions [16, 17]. A fluctuating system trapped inside a Josephson junction barrier will result in a fluctuating critical current via changes in the height of the tunneling barrier [18]. Other microscopic mechanisms could be the hopping of electrons in and out of microscopic traps in the oxide barrier [19, 20] and the hopping of magnetic fluxes between pinning sites [21].

The generic Hamiltonian accounting for this phenomenon should include not only the phase degree of freedom φ of the transmon, but also a collection of degrees of freedom $\{\chi\}$ that refer to the junction (for example the coordinates of the unstable atomic configurations),

$$\hat{H}(\varphi, \{\chi\}) = 4E_{\mathrm{C}}n^2 - E_{\mathrm{J}}(\{\chi\}) \cos\varphi + H_*(\{\chi\}), \tag{31}$$

where $\hat{H}_*(\{\chi\})$ designates the free Hamiltonian of the variables $\{\chi\}$. One has then to solve the Schrödinger equation for the wavefunction $|\Psi(\varphi, \{\chi\})\rangle$ of the entire system, comprising both the superconducting relative phase φ and the variables $\{\chi\}$.

In the absence of precise information about the dependence of the Hamiltonian on the variables $\{\chi\}$, we consider a simple model, which was used successfully in the literature [9], namely we presume that the dynamics of $\{\chi\}$ can be assimilated to a quasidegenerate two-level fluctuator with states $|\psi\rangle$ and $|\psi'\rangle$, corresponding to Josephson energies E_{J} and $E'_{\mathrm{J}} = E_{\mathrm{J}} + \delta E_{\mathrm{J}}$. The defect free Hamiltonian $\hat{H}_*(\{\chi\})$ can also introduce energy shifts with a characteristic scale $\hbar\omega_\tau$. These assumptions are sufficient to explain why the states should be split, as seen in the experiments. In this two-level approximation, the Hamiltonian reads

$$\hat{H}(\varphi) = 4E_{\mathrm{C}}n^2 - E_{\mathrm{J}} \cos\varphi |\psi\rangle\langle\psi| - E'_{\mathrm{J}} \cos\varphi |\psi'\rangle\langle\psi'| + \frac{\hbar\omega_\tau}{2} \left(|\psi\rangle\langle\psi| - |\psi'\rangle\langle\psi'|\right), \tag{32}$$

or, if we introduce the z-Pauli operator $\tau_z = |\psi\rangle\langle\psi| - |\psi'\rangle\langle\psi'|$ we can write the equation above as

$$\hat{H} = E_{\rm C} n^2 - E_J \cos\varphi - \frac{\delta E_{\rm J}}{2}(\mathcal{I} - \tau_z)\cos\varphi + \frac{\hbar\omega_\tau}{2}\tau_z, \tag{33}$$

where $\hat{\mathcal{I}} = |\psi\rangle\langle\psi| + |\psi'\rangle\langle\psi'|$ is the identity operator. Next, we diagonalize the transmon for each junction state $|\psi\rangle$ and $|\psi'\rangle$ separately, and find two sets of eigenenergies ω_j and ω_j' corresponding to $E_{\rm J}$ and $E_{\rm J'}$. Since we do not know exactly the value of ω_τ we assume that the splitting in the ground state can be neglected. If we work at the flux sweet point (minimum of the transition frequency in Supplementary Figure 4) the $h \times 27.5$ MHz shift in the Josephson energy of one of the junctions produces (see Ref. [1]) a shift of equal value $\Delta E_{\rm J} = h \times 27.5$ MHz in the effective Josephson energy of the device at this point, which is seen as a split of $\Delta \approx 11.5$ MHz in the frequency of the first transition. The model becomes then very similar to that discussed in Ref. [7], yielding in general a 6×6 matrix Hamiltonian. There are now many transitions possible, but since we do not know the details of how the field interacts with this rather complex system, we simply select those that are best resolved spectroscopically, and denote them by $|0\rangle, |1\rangle, |1'\rangle, |2\rangle$. The additional transitions, although covered by the broadened linewidth, are still somewhat visible in the spectrum of the first transition (see Supplementary Figure 4), as predicted by this model. For the simulations of Fig. 6 in the main text, the states $|1\rangle$, and $|1'\rangle$ are assumed to couple with equal strengths to the fields, in other words $\Omega_{01}(t) \approx \Omega_{01'}(t)$ and $\Omega_{12}(t) \approx \Omega_{1'2}(t)$. This empirical procedure is certainly a crude simplification of the actual situation, but we find indeed that the truncated Hamiltonian Eq. (28) is sufficient to reproduce the data. Finally, a consequence of the model above is that also the second excited state should spit. As we mentioned in the beginning, the absence of shifts along the two-photon detuning axis shows that it is enough to consider a single state $|2\rangle$. However, for larger powers we do observe much more complicated patterns, including shifts along the two-photon axis, which shows that the additional lines of the second excited states are coupled as well.

Supplementary References

[1] Koch, J. *et al.* Charge insensitive qubit design derived from the Cooper pair box. *Phys. Rev. A* **76**, 042319 (2007). URL http://journals.aps.org/pra/abstract/10.1103/PhysRevA.76.042319.

[2] Schuster, D. I. *et al.* ac Stark shift and dephasing of a superconducting qubit strongly coupled to a cavity field. *Phys. Rev. Lett.* **94**, 123602 (2005). URL http://link.aps.org/doi/10.1103/PhysRevLett.94.123602.

[3] Wallraff, A. *et al.* Strong coupling of a single photon to a superconducting qubit using circuit quantum electrodynamics. *Nature* **431**, 162–167 (2004). URL http://dx.doi.org/10.1038/nature02851.

[4] Bianchetti, R. *et al.* Dynamics of dispersive single-qubit readout in circuit quantum electrodynamics. *Phys. Rev. A* **80**, 043840 (2009). URL http://link.aps.org/doi/10.1103/PhysRevA.80.043840.

[5] Bianchetti, R. *et al.* Control and tomography of a three level superconducting artificial atom. *Phys. Rev. Lett.* **105**, 223601 (2010). URL http://link.aps.org/doi/10.1103/PhysRevLett.105.223601.

[6] Møller, D., Madsen, L. B. & Mølmer, K. Geometric phase gates based on stimulated Raman adiabatic passage in tripod systems. *Phys. Rev. A* **75**, 062302 (2007). URL http://journals.aps.org/pra/abstract/10.1103/PhysRevA.75.062302.

[7] Vogt, N., Cole, J. H., Marthaler, M. & Schön, G. Influence of two-level fluctuators on adiabatic passage techniques. *Phys. Rev. B* **85**, 174515 (2012). URL http://link.aps.org/doi/10.1103/PhysRevB.85.174515.

[8] Risté, D. *et al.* Millisecond charge-parity fluctuations and induced decoherence in a superconducting transmon qubit. *Nat. Commun.* **4**, 1913 (2013). URL http://dx.doi.org/10.1038/ncomms2936.

[9] Simmonds, R. W. *et al.* Decoherence in Josephson phase qubits from junction resonators. *Phys. Rev. Lett.* **93**, 077003 (2004). URL http://link.aps.org/doi/10.1103/PhysRevLett.93.077003.

[10] Gunnarsson, D. *et al.* Dielectric losses in multi-layer Josephson junction qubits. *Superconductor Science and Technology* **26**, 085010 (2013). URL http://stacks.iop.org/0953-2048/26/i=8/a=085010.

[11] Grabovskij, G. J., Peichl, T., Lisenfeld, J., Weiss, G. & Ustinov, A. V. Strain tuning of individual atomic tunneling systems detected by a superconducting qubit. *Science* **338**, 232–234 (2012). URL http://www.sciencemag.org/content/338/6104/232.abstract.

[12] Bal, M., Ansari, M. H., Orgiazzi, J.-L., Lutchyn, R. M. & Lupascu, A. Dynamics of parametric fluctuations induced by quasiparticle tunneling in superconducting flux qubits. *Phys. Rev. B* **91**, 195434 (2015). URL http://journals.aps.org/prb/abstract/10.1103/PhysRevB.91.195434.

[13] Lupaşcu, A., Bertet, P., Driessen, E. F. C., Harmans, C. J. P. M. & Mooij, J. E. One- and two-photon spectroscopy of a flux qubit coupled to a microscopic defect. *Phys. Rev. B* **80**, 172506 (2009). URL http://link.aps.org/doi/10.1103/PhysRevB.80.172506.

[14] Anderson, P., Halperin, B. I. & Varma, C. M. Anomalous low-temperature thermal properties of glasses and spin glasses. *Philosophical Magazine* **25**, 1–9 (1972). URL http://www.tandfonline.com/doi/abs/10.1080/14786437208229210.

[15] Phillips, W. A. Tunneling states in amorphous solids. *Journal of Low Temperature Physics* **7**, 351–360 (1972). URL http://link.springer.com/article/10.1007\%2FBF00660072.

[16] DuBois, T. C., Per, M. C., Russo, S. P. & Cole, J. H. Delocalized oxygen as the origin of two-level defects in Josephson junctions. *Phys. Rev. Lett.* **110**, 077002 (2013). URL http://link.aps.org/doi/10.1103/PhysRevLett.110.077002.

[17] DuBois, T. C., Russo, S. P. & Cole, J. H. Atomic delocalization as a microscopic origin of two-level defects in Josephson junctions. *New Journal of Physics* **17**, 023017 (2015). URL http://stacks.iop.org/1367-2630/17/i=2/a=023017.

[18] Ku, L.-C. & Yu, C. C. Decoherence of a Josephson qubit due to coupling to two-level systems. *Phys. Rev. B* **72**, 024526 (2005). URL http://link.aps.org/doi/10.1103/PhysRevB.72.024526.

[19] Rogers, C. T. & Buhrman, R. A. Composition of $\frac{1}{f}$ noise in metal-insulator-metal tunnel junctions. *Phys. Rev. Lett.* **53**, 1272–1275 (1984). URL http://link.aps.org/doi/10.1103/PhysRevLett.53.1272.

[20] Rogers, C. T. & Buhrman, R. A. Nature of single-localized-electron states derived from

tunneling measurements. *Phys. Rev. Lett.* **55**, 859–862 (1985). URL http://link.aps.org/doi/10.1103/PhysRevLett.55.859.

[21] Van Harlingen, D. J. *et al.* Decoherence in Josephson-junction qubits due to critical-current fluctuations. *Phys. Rev. B* **70**, 064517 (2004). URL http://link.aps.org/doi/10.1103/PhysRevB.70.064517.
